# Patient safety culture in nurses’ clinical practice

**DOI:** 10.1590/1518-8345.6231.3837

**Published:** 2023-03-27

**Authors:** Cláudia Patrícia da Costa Brás, Manuela Maria Conceição Ferreira, Maria do Céu Aguiar Barbieri de Figueiredo, João Carvalho Duarte

**Affiliations:** 1 Escola Superior de Enfermagem de Coimbra, Unidade de Investigação em Ciências da Saúde (UICISA: E), Coimbra, PT, Portugal.; 2 Escola Superior de Saúde de Viseu - Instituto Politécnico de Viseu, Unidade de Investigação em Ciências da Saúde (UICISA: E/ESEnfC - ESSV/IPV), Viseu, PT, Portugal.; 3 Universidade de Huelva, Departamento de Enfermagem, Spain.; 4 Escola Superior de Enfermagem do Porto CINTESIS/ESEP, Porto, PT, Portugal.

**Keywords:** Nurses, Professional Practice, Patient Safety, Safety Management, Organizational Culture, Hospitals, Enfermeiros, Prática Profissional, Segurança do Paciente, Gestão da Segurança, Cultura Organizacional, Hospitais, Enfermeros, Práctica Profesional, Seguridad del Paciente, Gestión de la Seguridad, Cultura Organizacional, Hospitales

## Abstract

**Objective:**

to assess the psychometric characteristics of the *Hospital Survey on Patient Safety Culture*, to characterize the patient safety culture, and to assess the influence of the sociodemographic and professional variables on the safety culture dimensions.

**Method:**

a methodological, observational, analytical and cross-sectional study conducted with 360 nurses in which the *Hospital Survey on Patient Safety Culture* questionnaire was used. The data were submitted to descriptive and inferential analysis, as well as to feasibility and validity studies.

**Results:**

the nurses’ mean age is 42 years old, their mean time of professional experience is 19 years, and they are mostly female. Good internal consistency was obtained (Cronbach’s alpha: 0.83), as well as acceptable model fit quality indices. Teamwork within units, Supervisor expectations and Feedback and communication about errors were the dimensions that obtained scores above 60%. Non-punitive response to error, Frequency of events reported, Support for patient safety and Staffing presented scores below 40%. These dimensions are influenced by age, schooling level and professional experience.

**Conclusion:**

the psychometric properties of the questionnaire certify its good quality. Teamwork can be considered as an enhancing factor for the safety culture. Assessing the safety culture allowed identifying problematic dimensions, thus enabling planning of future interventions.

## Resumo

**Objetivo:**

avaliar as caraterísticas psicométricas do *Hospital Survey on Patient Safety Culture,* caracterizar a cultura de segurança do doente e avaliar a influência das variáveis sociodemográficas e profissionais nas dimensões da cultura de segurança.

**Método:**

estudo metodológico, observacional, analítico, transversal, realizado com 360 enfermeiros, utilizando o questionário *Hospital Survey on Patient Safety Culture*. Os dados foram submetidos à análise descritiva, inferencial e estudos de fiabilidade e validade.

**Resultados:**

os enfermeiros possuem em média 42 anos de idade, 19 anos de experiência profissional e são maioritariamente do sexo feminino. Obteve-se boa consistência interna (alfa de Cronbach - 0,83) e índices aceitáveis de qualidade de ajustamento do modelo. O trabalho em equipa dentro das unidades, expetativas do supervisor, *feedback* e comunicação sobre o erro, foram dimensões que apresentaram scores acima dos 60%. A resposta ao erro não punitiva, frequência da notificação, apoio à segurança pela gestão, dotação de profissionais, apresentaram scores abaixo dos 40%. Estas dimensões são influenciadas pela idade, escolaridade e experiência profissional.

**Conclusão:**

as propriedades psicométricas do questionário certificam a sua qualidade. O trabalho em equipa pode ser considerado um fator potenciador da cultura de segurança. Avaliar a cultura de segurança permitiu identificar dimensões problemáticas, possibilitando o planeamento de intervenções futuras.

## Resumen

**Objetivo:**

evaluar las características psicométricas de la *Hospital Survey on Patient Safety Culture*, caracterizar la cultura de seguridad del paciente y evaluar la influencia de variables sociodemográficas y profesionales en las dimensiones de la cultura de seguridad.

**Método:**

estudio metodológico, observacional, analítico, transversal, realizado con 360 enfermeros, utilizando el cuestionario *Hospital Survey on Patient Safety Culture*. Los datos fueron sometidos a análisis descriptivo e inferencial y estudios de confiabilidad y validez.

**Resultados:**

los enfermeros tienen en promedio 42 años de edad, 19 años de experiencia profesional y la mayoría es de sexo femenino. Se obtuvo buena consistencia interna (alfa de Cronbach - 0,83) e índices aceptables de bondad de ajuste del modelo. El trabajo en equipo dentro de las unidades, las expectativas del supervisor, el *feedback* y la comunicación del error fueron las dimensiones que presentaron puntajes superiores al 60%. La respuesta al error no punitiva, la frecuencia de notificación, el apoyo a la seguridad por parte de los gestores, la dotación de personal, presentaron puntajes por debajo del 40%. Estas dimensiones son influenciadas por la edad, la educación y la experiencia profesional.

**Conclusión:**

las propiedades psicométricas del cuestionario certifican su calidad. El trabajo en equipo puede considerarse un factor que potencia la cultura de seguridad. La evaluación de la cultura de seguridad permitió identificar las dimensiones que tenían problemas y poder planificar futuras intervenciones.

**Highlights**

(1) Teamwork stands out as a factor that reinforces the safety culture.

(2) Under-reporting and a punitive culture predominate in health organizations.

(3) The professional experience enables a positive perception of the safety culture.

(4) The safety culture analysis allows planning interventions to reduce errors.

## Introduction

Patient safety is still one of the major challenges for 21^st^ century health institutions, whose main mission consists in providing good quality care. It is assumed that care provision is a risk activity due to its complexity, context and available resources, involving the possibility of uncertain and undesirable events^1^. Some studies show that a mean of 1 out of every 10 patients undergoes an adverse event in terms of receiving hospital care^2^. The 2021-2026 national plan for patient safety in Portugal reinforced the importance of promoting patient safety in a coordinated and persistent effort by all managers, mid-level leaders and health professionals to improve public awareness towards patient safety topics^3^. It is fundamental to analyse the real problems related to safe practices in care environments and the *Hospital Survey on Patient Safety Culture* (HSOPSC), developed by the Agency for Healthcare Research and Quality (AHRQ), allows assessing the safety culture, by measuring multiple dimensions related to values, beliefs, organizational norms, reporting of adverse events, communication, leadership and management^4^. The safety culture assessment allows diagnosing the safety culture, identifying areas for improvement, monitoring its evolution over time, enabling internal and external benchmarking to improve health care quality, and implementing change processes^5^.

Promoting patient safety is not a competitive mission for a single health system but a collaborative effort in which all systems must participate, and this collaboration considers a comparison of the safety culture across countries^6^. The HSOPSC Comparative Database is the only central repository to enable a data comparison with regard to evaluating the patient safety culture^4^. A routine assessment of the safety culture with disclosing and dissemination of the results at the institution level, planning improvement actions with the leaders’ and managers’ support, multifaceted programs and training were interventions that allowed six hospitals to improve their safety culture levels^7^.

Over the last few years, we have witnessed changes at an organizational level and in terms of Portuguese nurses’ practices, but we are still not at the desired safety culture level. If we check the data from the safety culture assessment at the national level that was performed in 2018, we find that some dimensions still need urgent intervention, specifically Frequency of events reported, Staffing and Non-punitive response to error^8^. If we refer to the evaluation of aspects related to the Portuguese national plan for patient safety until 2020, we find that there are communication failures between the various structures/departments of the health institutions, lack of involvement by professionals, specialists and patients in patient safety actions, lack of proximity and interaction between management, services and health professionals, and absence of legislation providing confidentiality and protection to the professionals involved in the notification of an adverse event^3^.

The change in culture in the health system largely depends on the involvement and participation of different employees, but particularly on nurses’, who represent the professional group that most interacts with patients due to their uninterrupted care practice. In this health care provision, the actors’ individual and/or social characteristics are crucial for the safety culture, knowing that, over time, the professionals acquire such culture through their participation and coexistence in the organizational environment^9^. In some studies, age, gender, work experience and schooling level were significant predictors of the nurses’ perceptions about the patient safety culture^10^ ^-^ ^11^.

Further studies that assess the patient safety culture from the nurses’ point of view are fundamental, so that current patient safety problems in health institutions can be more readily identified.

Developing a safety culture is hard work that does not occur automatically; it is a challenge, particularly in large hospitals, such as the one in question. It is essential to assemble organized and resilient work teams with strong communicative power and the ability to maintain actions, which respond and adapt to the pressure of the different clinical risks intrinsic to health care provision activities^12^ ^-^ ^13^. This study aimed at assessing the psychometric characteristics of HSOPSC, at characterizing the patient safety culture and at assessing the influence of the sociodemographic and professional variables on the safety culture dimensions.

## Method

### Type of study

This is a methodological, observational, analytical and cross-sectional study. The methodological study^14^ adopted the psychometric procedures as a framework^15^ ^-^ ^16^ to establish and verify reliability and validity of HSOPSC, according to the COSMIN^17^ (COnsensus-based standards for the Selection of health Measurement INstruments) protocol from the EQUATOR network. The observational study was guided by the Strengthening the Reporting of Observational Studies in Epidemiology (STROBE) tool^18^, which allowed assessing the patient safety culture and the influence of the sociodemographic and professional variables on the safety culture dimensions.

### Study locus

Data collection was developed in a hospital and university centre from the central region of Portugal. This hospital is an institution that belongs to the National Health Service, offering coverage to nearly 2,231,346 inhabitants living in the aforementioned region and providing high quality health services and differentiation. It has a quality and patient safety office, where a commission for such purposes operates, with the objective of consolidating a patient safety and risk management culture in line with the Strategy for Quality in Health in Portugal. In the scope of organizational clinical quality, the actions performed were centred on the integrated medication management system. To reinforce patient safety, training actions were carried out aimed at the areas of communication, patient involvement in their own safety, fire safety, basic precautions for infection control and health risk management. 

### Period

The data collection period was from September 2018 to May 2019.

### Population

The accessible population consisted of 2,891 nurses working in a hospital and university centre from the central region of Portugal.

### Selection criteria 

The inclusion criteria considered corresponded to all the nurses developing functions related to the provision of direct care to patients and who accepted to voluntarily participate in the study. The exclusion criteria established were as follows: performing functions as a nurse manager and being temporarily distanced from the service during the data collection period due to medical, vacations or other types of leaves.

### Definition of the sample

Non-probabilistic and convenience sample was used, comprised by 360 participants. Sample size calculation took into account the guidelines set forth in the user guide proposed by AHRQ, recommending a minimum response rate of 50%, depending on the minimum sample size and the population under study^19^. In this sense, for a population between 1,000 and 2,999 individuals, 300 subjects will be the minimum limit to attain the minimum response rate of 50%^20^. The sample under study is comprised by 360 participants, a number slightly higher than the recommended.

### Study variables

The patient safety culture dimensions were considered as the dependent variable and the sociodemographic variables (gender, age group, academic qualifications) and professional context variables (specialization title, specialization area and years of professional experience) were the independent variables.

### Instruments used for data collection

The instruments for data collection consisted of an *ad hoc* questionnaire that allowed the sociodemographic (gender, age, marital status, academic qualifications) and professional context (specialization degree, specialization area and years of professional experience) characterization, in addition to HSOPSC - version 1, created by AHRQ. This instrument has great potential to identify the safety culture determinants, to assess the professionals’ opinions about patient safety, error and event reporting, thus guiding continuous investment in patient safety^21^. The original version was translated, culturally adapted and validated for European Portuguese, involving all health professionals from three hospitals (nurses, physicians, medical assistants, senior technicians, administrative clerks, and diagnostic and therapeutic technicians)^22^. In this study, the population consists of Portuguese nurses; therefore, the psychometric assessment of the instrument is relevant to confirm model fit quality, corroborating the HSOPSC internal structure. 

HSOPSC encompasses forty-two items in the form of a *Likert*-type ordinal scale graduated into five levels: from 1 (I strongly disagree or Never) to 5 (I strongly agree or Always). It was designed to assess 12 safety patient dimensions, recommending the authors that, for analysing the results, these same dimensions should be grouped into three larger dimensions. The dimensions at the *level of the services/units* comprise the following: Dimension 1-Teamwork within the units (4 items), Dimension 2-Supervisor/Manager expectations and actions in promoting patient safety (4 items), Dimension 3-Organizational learning - Continuous improvement (3 items), Dimension 4-Management support for patient safety (3 items), Dimension 6-Feedback and communication about error (3 items), Dimension 7-Communication openness (3 items), Dimension 10-Staffing (4 items), and Dimension 12-Non-punitive response to error (3 items). The dimensions at the *hospital level* include Dimension 9-Teamwork across units (4 items) and Dimension 11-Handoffs and transitions (4 items). The safety culture *outcome variables* encompass Dimension 5-Overall perception of patient safety (4 items) and Dimension 8-Frequency of events reported (3 items). The questionnaire consists of two single-item variables that are assessed separately, without comprising the dimensions (Patient safety level and Number of events reported in the last 12 months)^19^.

### Data collection

The questionnaires were handed in paper format to the nurses, requesting their informed consent and reinforcing the anonymous and confidential nature of data treatment. Several informal visits were made to the services to collect the questionnaires, notice difficulties in their filling out and promote nurses’ participation in the research. The questionnaires were returned in a closed envelope, separately from the informed consent form. A total of 620 questionnaires were delivered, of which 360 were returned, representing an adherence rate of 58.0%, exceeding the researcher’s minimum goal, which would be 50% if we refer to the AHRQ criteria^19^.

### Data treatment and analysis

The methodology proposed in the AHRQ user’s guide^19^ was followed to analyse and interpret the HSOPSC results. The negatively formulated items were reversed (A5, A7, A8, A10, A12, A14, A16, A17, B3, B4, C6, F2, F3, F5, F6, F7, F9, F11). The authors of the scale recommend that, in order to ease data analysis, all five answer levels of the original *Likert*-type ordinal scale should be recoded into 3 answer levels (positive, neutral, negative). The percentage of positive answers corresponds to the combination of participants who answered “I strongly agree” or “I agree” or “Always” or “Most of the times”, depending on the answer categories used for each item. The level considered “neutral” consists of the midpoint of the scale, corresponding to the combination of “I don’t agree or disagree” or “Sometimes” answers. The percentage of negative answers corresponds to the combination of participants who answered “I strongly disagree” or “I disagree” or “Never” or “Rarely”, depending on the answer categories used for each item. The instrument items were grouped into safety culture dimensions according to AHRQ. The safety culture dimensions were calculated by determining the mean (unweighted) value of the classifications of the items by dimension. AHRQ considers that positive ratings equal to or greater than 75% designate dimensions that represent strong safety culture areas and that average values of positive answers equal to or less than 50% indicate dimensions that represent problem areas. The percentage of positive answers given to the items was considered by dividing the number of positive answers by the total number of answers (positive, neutral, negative)^23^.

Regarding the sociodemographic and professional characterization items, the data were analysed using descriptive statistics, encompassing a set of central tendency and dispersion measures. In order to identify the groups with statistical differences between each other, Mann-Whitney’s non-parametric U test was used, considering a Type I error of 5%^24^. The IBM^®^ SPSS^®^ Statistics for Windows software program, version 27.0 (IBM Corp., Armonk, N.Y., USA) was used for the statistical data analysis.

In this study, internal consistency of the items was determined by means of Cronbach’s alpha coefficient, adopting values above 0.7 as a reference for good internal consistency. In the analysis of the factor model, sensitivity of the items was verified through shape (asymmetry and kurtosis) and association (Pearson’s correlation) measures, according to the type of variable and to the measuring scale. Construct validity was performed through Confirmatory Factor Analysis (CFA) resorting to the AMOS^®^ 27 software (Analysis of Moment Structures)^25^. It was considered that asymmetry absolute values below 3 and flattening values below 7 do not compromise sensitivity of the models^26^. As an additional technique to verify the measuring quality, Composite Reliability (CR) and Mean Extracted Variance (MEV) were determined for each of the dimensions. As reference values, indices higher than 0.70 are suggested for CR, although lower values may be acceptable for exploratory research studies and, for MEV, values​greater than or equal to 0.50 are considered, indicators of adequate validity, with the possibility of flexibilizing this limit to 0.40^27^.

The following global fit quality indicators were used: ratio between chi-square and degrees of freedom (χ^2^/DoF), considering perfect fit if (χ^2^/DoF) is equal to 1, good when below 2, and acceptable when below 5. The GFI (Goodness of Fit Index) and CFI (Comparative Fit Index) fit quality indices are considered good when above 0.90. RMSEA (Root Mean Square Error of Approximation), RMR (Root Mean Residual) and SRMR (Standardized Root Mean Square Residual) are considered adequate when below 0.08^28^.

### Ethical aspects

The research protocol was submitted to and approved by the Ethics Commission integrated in the Innovation and Development unit - Clinical Trials centre and by the Board of Directors of the hospital and university centre in the central region of Portugal, having obtained formal authorization for continuation of the study - Registration No. 8,742/2017. An authorization request was made to the author who was in charge of validating HSOPSC for the Portuguese population. 

## Results

The study sample mostly consists of female nurses (82.8%), with a mean age of 42 years old. Most of the nurses are married or live in a *de facto* union (63.6%). The nurses’ predominant academic qualifications correspond to Nursing Bachelor’s Degree (78.6%) and only 38.3% have a Specialized Nurse professional degree. Of them, 50.4% are specialized in Maternal Health and Obstetric Nursing (MHON) and 49.6% are specialized in other Nursing areas. Altogether, the nurses have a mean of 19 years of professional experience. 

### Assessment of the psychometric properties of the HSOPSC instrument

All the HSOPSC items were tested by resorting to CFA. Once sensitivity of the items was analysed, it was observed that, in general, the asymmetry and kurtosis absolute values do not compromise CFA performance^26^ ^,^ ^28^ since, in absolute values, they range between 0.069 and 1.253 for asymmetry and between 0.048 and 2.13 for kurtosis. 

As for the construct validity, most of the items have saturation values above 0.50 with the corresponding factor, except for items A16, A7, F4; and individual reliability of the items assumes indices greater than 0.25, except for item A7, although it was decided to maintain them according to the AHRQ guide original version^19^.

**Figure 1:**
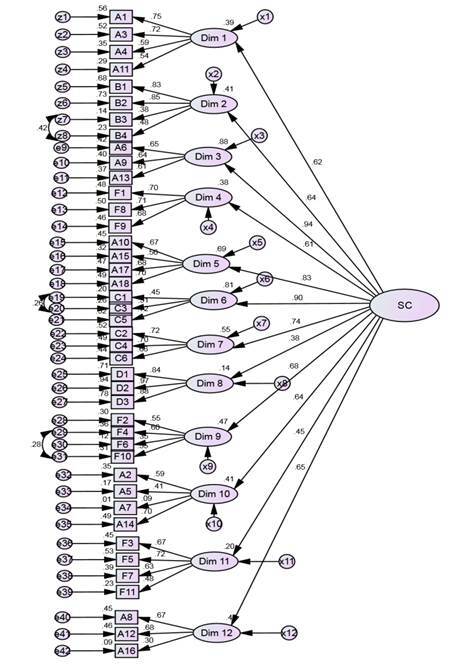
Second order hierarchical structure

The global goodness of fit indices in the first evaluation presented good fit for χ^2^/DoF=1.990, RMR=0.053, SRMR=0.059 and RMSEA=0.053 and poor fit for GFI=0.830 and CFI=0.861. The model was re-specified through the modification indices proposed by the AMOS program, obtaining global fit indices indicative of an adjusted model with values of χ^2^/DoF=1.842; RMR=0.053, SRMR=0.058 and RMSEA=0.048, remaining tolerable for GFI=0.844; CFI=0.882^28^. Considering that most of the factors presented high correlational values, it is assumed that these correlations suggest the existence of a 2^nd^ order factor. Thus, a hierarchical structure with a 2^nd^ order factor is proposed, designated as “Safety Culture” (SC). The global fit quality values remain with slight differences in relation to the aforementioned ones: χ^2^/DoF=2.176, RMR=0.064, SRMR=0.072, RMSEA=0.057, GFI=0.807 and CFI=0.824. We notice that the indices represent good fit of the model in relation to χ^2^/DoF, RMR, SRMR and RMSEA and tolerable fit for GFI and CFI, a fact that may be related to the sample size^28^. The dimensions that best explain the safety culture construct are “Organizational learning - Continuous improvement”, “Overall perception of patient safety”, “Feedback and communication about errors” and “Communication openness”, as illustrated in Figure 1.

An internal reliability or consistency analysis was performed, evaluated by means of Cronbach’s alpha (α). With all twelve patient safety dimensions, coefficients varying from 0.49 (weak) to 0.93 (very good) were observed, values very close to the scale validation study in Portugal^22^. The global score of this instrument revealed good internal consistency (α=0.832)^29^ ^-^ ^30^. It was also verified that the dimensions under study do not present normal distribution (p>0.05), as shown in Table 1.

**Table 1:** Cronbach’s alpha values and statistics related to all twelve patient safety dimensions of the HSOPSC instrument^*^ for the 360 nurses. Coimbra, PT, Portugal, 2018-2019

	No. of items	M^†^ (±SD^‡^)	α^§^ Current study (2019)	α Study by Eiras, et al. (2014)	*p-value* ^||^
*Dimensions at the level of the services/units*					
Teamwork within units	4	3.73 (±0.62)	0.73	0.73	0.001
Supervisor/Manager expectations and actions promoting patient safety	4	3.69 (±0.67)	0.75	0.72	0.001
Organizational learning - Continuous improvement	3	3.53 (±0.64)	0.66	0.71	0.001
Management support for patient safety	3	2.87 (±0.76)	0.73	0.62	0.001
Feedback and communication about errors	3	3.56 (±0.65)	0.64	0.76	0.001
Communication openness	3	3.47 (±0.70)	0.73	0.67	0.001
Staffing	4	2.96 (±0.69)	0.49	0.48	0.001
Non-punitive response to error	3	2.86 (±0.69)	0.57	0.57	0.001
*Dimensions at the hospital level*					
Teamwork across units	4	3.15 (±0.55)	0.61	0.69	0.001
Handoffs and transitions	4	3.35 (±0.65)	0.72	0.71	0.001
*Patient safety outcome variables*					
Overall perception of patient safety	4	3.22 (±0.78)	0.75	0.62	0.001
Frequency of events reported	3	2.67 (±1.02)	0.93	0.90	0.001
*Total items*	42		0.83	0.91	

*HSOPSC = *Hospital Survey on Patient Safety Culture*; ^†^M = Mean; ^‡^SD = Standard Deviation; ^§^α = Cronbach’s alpha; ^||^p = Kolmogorov-Smirnov test (p-value<0.001)

The values of the scale’s variables and the correlations between all twelve dimensions were evaluated by means of Pearson’s Correlation Matrix. The results obtained indicate positive and significantly correlated dimensions, except for the correlation between dimension 8 and dimensions 10, 11 and 12, which is not significant.

### Characterization of the safety culture by the nurses

The presence of dimensions with percentages of positive answers varying from 22.6% to 70.5% is verified. None of the dimensions reached scores above 75% as indicated by the authors of the scale^23^. The “Teamwork within units”, “Supervisor/Manager expectations and actions promoting patient safety” and “Feedback and communication about error” dimensions assume higher positive percentages (between 64.8% and 70.5%). Seven dimensions obtained percentages of positive answers below 50%, as shown in Table 2.

**Table 2:** Mean percentage of negative, neutral and positive answers given by all 360 nurses to the Patient Safety Culture dimensions. Coimbra, PT, Portugal, 2018-2019

Patient safety culture dimensions	Mean % of negative answers	Mean % of neutral answers	Mean % of positive answers
*Dimensions at the level of the services/units*			
Teamwork within units	9.8	19.7	70.5
Supervisor/Manager expectations and actions promoting patient safety	11.0	23.8	65.2
Organizational learning - Continuous improvement	11.5	31.2	57.3
Management support for patient safety	35.9	36.3	27.8
Feedback and communication about error	13.7	21.5	64.8
Communication openness	16.0	26.3	57.7
Staffing	39.6	23.3	37.1
Non-punitive response to error	39.5	33.9	26.6
*Dimensions at the hospital level*			
Teamwork across units	26.0	40.9	33.1
Handoffs and transitions	19.5	31.4	49.1
*Patient safety outcome variables*			
Overall perception of patient safety	26.6	27.5	45.9
Frequency of events reported	45.3	32.1	22.6

Regarding the assessment of the patient safety level in the service/unit, it was found that 50.8% of the nurses considered it *acceptable*, 36,9% considered it *very good,* 10.5% considered it *weak* or *very weak* and 1.7%% thought of it as *excellent.*

Regarding the Frequency of events reported, it was verified that most of the nurses (79.4%) had not notified any event in the last 12 months. 10.6% of the participants had notified from 3 to 5 events and 8.9% of the nurses had reported only 1 to 2 events. 

### Relationship of the sociodemographic and professional variables with the patient safety culture dimensions

As for the sociodemographic variables, no statistically significant differences are recorded between the gender variable in relation to the safety culture dimensions; however, if we consider a 10% significance level (p<0.10), we can consider that the results are marginally significant^27^. For Dimension 10, male nurses present higher mean values. Regarding the age group, statistical significance was found in dimensions 2, 3, 5 and 11, verifying that the mean values are higher in the nurses aged at least 40 years old. The academic qualifications exert an influence on dimensions 3, 5, 6 and 8, with nurses who have a Bachelor’s degree presenting the highest mean values, as shown in Table 3.

**Table 3:** Association between the nurses’ sociodemographic variables (gender, age group, academic qualifications) and the patient safety culture dimensions. Coimbra, PT, Portugal, 2018-201

Variable	Gender			Age group			Academic qualifications		
	Female	Male	p-value^*^	< 40 years old	> 40 years old	p-value^*^	BD^†^	M/P^‡^	p-value^*^
	Mean value			Mean value			Mean value		
Dim. 1^§^	180.8	179.0	0.897	171.3	188.3	0.118	183.5	166.8	0.242
Dim. 2^||^	178.8	188.6	0.496	166.1	192.7	0.015	182.6	170.7	0.402
Dim. 3^¶^	181.5	175.6	0.679	167.0	191.9	0.022	186.9	150.8	0.011
Dim. 4^**^	180.7	179.8	0.95	171.9	187.8	0.147	180.0	182.9	0.839
Dim. 5^††^	180.2	182.2	0.889	164.0	194.4	0.005	187.3	149.1	0.007
Dim. 6^‡‡^	180.0	182.9	0.835	172.3	187.5	0.157	189.1	140.7	0.001
Dim. 7^§§^	179.5	185.4	0.676	181.1	180.0	0.918	180.6	180.1	0.971
Dim. 8^||||^	179.7	184.4	0.741	175.9	184.4	0.432	188.3	144.5	0.002
Dim. 9^¶¶^	178.7	189.3	0.46	173.0	186.9	0.202	184.7	161.2	0.099
Dim. 10^***^	175.7	203.7	0.052	170.2	189.2	0.081	184.7	160.9	0.095
Dim. 11^†††^	181.0	178.2	0.844	167.0	191.9	0.022	181.8	174.4	0.603
Dim. 12^‡‡‡^	179.5	185.3	0.686	181.3	179.8	0.892	177.5	194.6	0.227

*Mann-Whitney test, p-value<0.05; ^†^BD = Bachelor’s Degree; ^‡^M/P = Master’s Degree/PhD; ^§^Dim. 1 = Teamwork within units; ^||^Dim. 2 = Supervisor/Manager expectations and actions promoting patient safety; ^¶^Dim. 3 = Organizational learning - Continuous improvement; ^**^Dim. 4 = Management support for patient safety; ^††^Dim. 5 = Overall perception of patient safety; ^‡‡^Dim. 6 = Feedback and communication about errors; ^§§^Dim. 7 = Communication openness; ^||||^Dim. 8 = Frequency of events reported; ^¶¶^Dim. 9 = Teamwork across units; ^***^Dim. 10 = Staffing; ^†††^Dim. 11 = Handoffs and transitions; ^‡‡‡^Dim. 12 = Non-punitive response to error

Regarding the professional variables, statistically significant differences were identified in dimensions 3, 5 and 8 with regard to the existence of a specialization degree, verifying that the mean values are higher in the nurses who do not have any specialization degree. Regarding the specialization area, statistical significance was found in dimensions 2, 4 and 12, with the nurses specialized in MHON presenting the lowest mean values in these dimensions in relation to those from other Nursing specialization areas. Professional experience exerts a significant influence on dimensions 2, 4, 3 and 5. Nurses who have more than 20 years of professional experience have higher mean values in the aforementioned dimensions than those with fewer years of professional experience, as shown in Table 4.

**Table 4:** Association between the nurses’ (n=360) professional variables (specialization degree, specialization area, professional experience) and the patient safety culture dimensions. Coimbra, PT, Portugal, 2018-2019

Variable	Specialization degree			Specialization area			Professional experience		
	No	Yes	p-value^*^	^†^ **MHON**	Other area	p-value^*^	< 20 years	> 20 years	p-value^*^
	Mean value			Mean value			Mean value		
Dim. 1^‡^	176.5	186.9	0.353	74.1	65.8	0.217	179.6	181.8	0.841
Dim. 2^§^	180.5	180.6	0.995	62.7	77.4	0.03	170.2	194.7	0.026
Dim. 3^||^	191.3	163.4	0.012	65.9	74.1	0.222	168.1	197.7	0.007
Dim. 4^¶^	178.8	183.3	0.688	57.9	82.3	0.001	170.4	194.5	0.028
Dim. 5^**^	191.6	162.8	0.01	65.9	74.1	0.228	167.3	198.8	0.004
Dim. 6^††^	187.4	169.6	0.106	65.6	74.4	0.191	173.0	190.9	0.099
Dim. 7^‡‡^	178.1	184.4	0.572	67.9	72.2	0.525	176.1	186.6	0.334
Dim. 8^§§^	195.2	157.2	0.001	67.0	73.0	0.371	174.8	188.5	0.213
Dim. 9^||||^	178.1	184.4	0.573	74.7	65.3	0.165	175.0	188.1	0.236
Dim. 10^***^	179.3	182.4	0.778	66.1	74.0	0.247	171.8	192.6	0.059
Dim. 11^†††^	172.5	193.2	0.063	71.1	68.9	0.743	173.6	190.1	0.134
Dim. 12^‡‡‡^	178.7	183.4	0.675	56.7	83.5	0.001	178.3	183.6	0.629

*Mann-Whitney test, p-value<0.05; ^†^Maternal Health and Obstetric Nursing; ^‡^Dim. 1 = Teamwork within units; ^§^Dim. 2 = Supervisor/Manager expectations and actions promoting patient safety; ^||^Dim. 3 = Organizational learning - Continuous improvement; ^¶^Dim. 4 = Management support for patient safety; ^**^Dim. 5 = Overall perception of patient safety; ^††^Dim. 6 = Feedback and communication about errors; ^‡‡^Dim. 7 = Communication openness; ^§§^Dim. 8 = Frequency of events reported; ^||||^Dim. 9 = Teamwork across units; ^***^Dim. 10 = Staffing; ^†††^Dim. 11 = Handoffs and transitions; ^‡‡‡^Dim. 12 = Non-punitive response to error

## Discussion

The results point to a safety culture measuring instrument with satisfactory reliability, reaching a global Cronbach’s alpha of 0.83. Staffing and Non-punitive response to errors were the dimensions with lowest reliability, as was the case in the scale validation study^22^. Construct validity was considered adequate and all the dimensions were positively correlated. The global fit quality of the model is acceptable, maintaining the multidimensional structure of the initial questionnaire, which does not compromise comparability with other studies. 

In the current study, higher prevalence of female nurses was verified (82.8%), a fact that is corroborated by other studies in which the feminization rate is high among Nursing professionals and in other health professionals^31^ ^-^ ^32^.

It was found that none of the twelve safety culture dimensions reached the required percentage of positive answers to be considered as strong patient safety culture dimensions^23^. All dimensions present results below the mean positive percentage when compared to the results of the AHRQ Hospital Research Database dimensions^4^. However, the results of this study are quite similar to those obtained in the assessment of the safety culture at the national level and in the central region of Portugal^8^. 

Teamwork within units was the dimension that obtained the best results (71%), corroborated by other studies^33^ ^-^ ^35^, suggesting that this dimension may be a factor that enhances the safety culture^36^. Good interaction between the teams, mutual support and respect exert a positive impact on Nursing care^37^. However, teamwork across units was a weak area (33%), in line with the international literature^38^ ^-^ ^40^, which refers to lack of support and coordination between departments. 

The Supervisor/Manager expectations and actions promoting patient safety evidenced a positive mean of 65%, which allows assuming that nurses recognize the supervisor’s role in promoting patient safety. Similar results are found in international studies^41^ ^-^ ^43^, which prove that leaders promote a learning culture and raise awareness among the employees. The organizational culture is positively correlated with the leaders’ behaviours, due to their influence on the development of behaviours, values and beliefs in their employees^13^. This premise is also noticed in the results of the feedback and communication about error dimension (65%), results evidenced in some studies^36^ ^,^ ^42^ ^-^ ^43^, noting that professionals are informed about errors that occur and openly discuss ways to prevent them. Despite everything, it is important to continue investing in Communication openness, a dimension that obtained a positive mean of only 58%, a value certified by other international studies^33^ ^,^ ^35^ ^,^ ^44^, and which includes a culture of communication between different hierarchical levels. Improving the work processes through effective communication is an important tool for error prevention and should be encouraged to achieve care quality^33^. In this dimension, one of the items (“the professionals speak freely if they notice that something negatively affects patient care”) stands out, which allows deducing that nurses have a high sense of responsibility regarding patient safety, not ignoring existing problems. 

Despite this communication openness and feedback, nurses remain reluctant to report events. The Frequency of events reported dimension has a significantly low mean positive evaluation (23%), as in the case of national and international studies^8^ ^,^ ^45^. This result is understood by the high number of professionals in this study who did not report any event in the last 12 months. Also in an international study, half of the health professionals had never reported incidents related to patient safety during the last year^46^. Detection of the error and its immediate notification is crucial for the implementation of preventive interventions and measures in order to reduce the harms caused^40^. The main reasons reported by the health professionals for notifying events are related to pressure from managers, work overload, forgetfulness, devaluation of the error, lack of knowledge about how to report, and lack of feedback from the notifications made^47^. Underreporting is frequently related to the Non-punitive response to error dimension, which also obtained one of the low positive mean values (27%), similarly to studies conducted in other countries^36^ ^,^ ^43^ ^,^ ^48^. The reduced frequency of reporting adverse events may suggest a punitive culture, which is still present in some health organizations^43^. This situation is also related to the fact that the notification system in Portugal, even if considered anonymous, does not safeguard non-identification of the professionals. As long as there is no legal regulation of the incident systems in Portugal, which ensure confidentiality and non-punishment for the notifications, reporting an adverse event can be used as evidence in a legal process and, in this way, underreporting will continue to be a reality, thus preventing organizational learning^49^.

Nurses consider that a learning culture where errors lead to positive changes is fundamental, highlighting the Organizational learning - Continuous improvement dimension with a positive mean of 57%, results identified in other studies^37^ ^,^ ^40^ ^,^ ^44^ ^,^ ^50^, in which culture monitoring activities and feedback on the safety results reinforce the professionals’ competence processes. 

The Overall perception of patient safety dimension has a positive mean of 46%, very close to the results obtained in international studies^36^ ^,^ ^43^ ^,^ ^44^ ^,^ ^51^. Nurses perceive the high-risk nature of health organizations that leads to the occurrence of incidents resulting from a sequence of systemic factors, which include the organization’s strategies, culture, work practices and risk prevention^13^; therefore, they consider that hospital management should prioritize patient safety. However, the Management support for patient safety dimension obtained a positive mean of 28%, evidencing that nurses consider that there is still little commitment and support by the hospital management, data which are similar to other studies^52^ ^-^ ^53^, where the hospital management does not provides an environment of trust and motivation in the workplace. The Staffing dimension was also considered a weak area in the safety culture assessment, with a low positive mean (37%), a value that is also low in several international hospitals^36^ ^,^ ^44^. Most of the nurses from a hospital in Sweden mentioned that work was often performed with reduced staff, causing fatigue and exhaustion that exerted an impact on the quality of patient care^36^. The Handoffs and transitions dimension reached a positive mean of 49%, which is in line with other studies^33^ ^,^ ^44^ ^-^ ^45^, evidencing a safety culture area that also needs improvement. Due to fragmentation of the health systems, nurses are faced with an increase in the number of care transfers, with a consequent greater probability of communication failures. 

From the results found, it is verified that gender only has explanatory power on the patient safety culture, with regard to the “Staffing” dimension, which allows inferring that male Nursing professionals appear to have a more positive perception of the safety culture in terms of this dimension. Divergences were detected in a study found^10^, which states that female Nursing professionals revealed a better safety culture.

In this study, both the nurses with more than 20 years of professional experience and those aged at least 40 years old state having a more positive perception of the safety culture in the “Supervisor/Manager expectations and actions promoting safety” “Organizational learning - Continuous improvement” and “Overall perception of patient safety” dimensions. According to the international literature, it was verified that older nurses had a better perception of patient safety than younger ones and that, as nurses’ years of experience increased, the overall perception of patient safety increased accordingly^11^. It was found that nurses aged between 40 and 60 have a more positive view of the patient safety culture, having a better understanding of the patient’s needs^46^. These results may be related to the fact that more experienced professionals are more likely to detect risks and feel more confident in revealing their true perceptions^40^. Also in a Brazilian university hospital, higher age and time of professional experience were associated with better perceptions of the patient safety culture in the “Supervisor/Manager expectations and actions promoting patient safety”, “Organizational learning - Continuous improvement” dimensions^53^. It was found that nurses with a Bachelor’s degree, as well as those without any specialization degree, evidence a more positive perception of the safety culture in the “Organizational learning - Continuous improvement”, “Overall perception of patient safety” and “Frequency of events reported” dimensions. The results also showed that nurses with a Bachelor’s degree level present a more positive perception of the safety culture with regard to the “Feedback and communication about error” dimension. These data suggest that nurses with lower academic levels are willing to improve their knowledge, seeking more differentiation of contents in the patient safety context. A study revealed that the “Frequency of events reported” dimension was significantly different according to the academic qualifications, being inversely proportional to schooling level^53^. However, data were found revealing that health professionals with a Master’s degree and better educational status obtained higher patient safety culture scores than those with a Bachelor’s degree as academic qualification^10^.

Nurses from other Nursing specialization areas stated having a more positive perception of the safety culture in the “Supervisor/Manager expectations and actions promoting patient safety”, “Management support for patient safety” and “Non-punitive response to error” dimensions than those in the MHON areas. The results of this dimension suggest that MHON nurses notice weaknesses regarding the hospital manager and management support in the promotion of patient safety. Divergences were found in a study^54^, which states that nurses from the maternal and child service revealed safety culture aspects that were stronger than in the medical clinic and surgical clinic services. 

The following limitations of the current study are identified: the fact that the sample is not probabilistic, which conditions data representativeness, making it impossible to extrapolate the results to other samples. In addition, the safety culture assessment may include the use of qualitative methods to enable a deeper understanding of nurses’ perceptions and provide insights into areas for improvement. Despite the limitations presented, this study brings about important contributions to the knowledge about the patient safety culture, which will serve as a work basis to develop actions that ensure safe Nursing care in health institutions. This study provided important data that may be useful to discuss the topic in the study plans of the Bachelor’s degree in Nursing, as well as in the MSc and graduate courses in Nursing. 

## Conclusion

The psychometric characteristics of HSOPSC allow asserting that this instrument is a tool that makes it possible to consistently assess the patient safety culture, providing an adequate structure for what is intended to be measured.

The patient safety culture from the nurses’ perspective was evaluated containing dimensions with values close to being able to be considered enhancers of a positive safety culture (Teamwork within units, Supervisor/manager expectations and actions and Feedback and communication about error). The Organizational learning - Continuous improvement, Communication openness, Handoffs and transitions, Overall perception of patient safety dimensions are also positively valued, although they should be subjected to some intervention so that they can be considered strong aspects of the safety culture. On the other hand, it was found that there are five dimensions considered problematic, with a need for priority intervention (Non-punitive response to error, Frequency of events reported, Management support for patient safety, Staffing and Teamwork across units). The multiple dimensions have the ability to influence each other and are also influenced by age, professional experience and academic qualifications. 

This study reveals that it is essential to continually assess the safety culture to diagnose areas for improvement in order to promote patient care quality and safety. The study data expose weak areas from one of the health institutions in Portugal, a scenario that is identical at the national and international level, and which commit hospital management, managers and leaders to establish approaches and improvement methods to reduce the impact on patient safety. Interventions are suggested in the dimensions considered as weaker, in order to provide a clinical practice environment with greater cooperation between hospitals and with adequate staffing, improving information feedback and encouraging the notification of events to promote safer health care for patients and health professionals.
